# Knowledge and Use of Menthol-Mimicking Cigarettes Among Adults in the US

**DOI:** 10.1001/jamanetworkopen.2024.54608

**Published:** 2025-01-14

**Authors:** Kelvin Choi, Kristen R. Hamilton-Moseley, Lilianna Phan, Ayesha Azeem, Bambi Jewett, Kasra Zarei, Kiana Hacker

**Affiliations:** 1 Division of Intramural Research, National Institute on Minority Health and Health Disparities, Bethesda, Maryland; 2 Department of Community Health and Prevention, Dornsife School of Public Health and Division of Graduate Nursing, College of Nursing and Health Professions, Drexel University, Philadelphia, Pennsylvania

## Abstract

**Importance:**

Cigarette companies have been introducing synthetic cooling agent menthol-mimicking cigarettes into the US marketplace as menthol cigarette bans are implemented. These cigarettes may reduce the public health benefits of menthol cigarette bans.

**Objective:**

To examine the epidemiology of the use of synthetic cooling agent menthol-mimicking cigarettes among adults in the US.

**Design, Setting, and Participants:**

This population-based cross-sectional survey study of adults in the US (≥21 years) with oversampling of Asian, American Indian or Alaska Native, Black or African American, and Hispanic or Latino peoples was conducted from March to May 2024. Participants were recruited from an online panel using a sample-matching approach to achieve national representation. Data were weighted to be nationally representative. Descriptive statistics were used to summarize the population characteristics. Weighted prevalence of awareness of, ever use (among those aware), current use (among ever used), susceptibility (among noncurrent use), and likely to switch to synthetic cooling agent menthol-mimicking cigarettes were estimated overall and by covariate. Sequential weighted logistic regression models were used to estimate the associations between covariates and synthetic cooling agent menthol-mimicking–related outcomes (awareness, ever use, current use, susceptibility). Statistical analysis was performed from July to September 2024.

**Exposures:**

Demographics, state menthol cigarette ban, cigarette smoking, and disclosure of synthetic cooling agent.

**Main Outcomes and Measures:**

Awareness of, susceptibility to, ever and currently using synthetic cooling agent menthol-mimicking cigarettes and intention to switch to these cigarettes if a nationwide menthol cigarette ban were enacted.

**Results:**

The study population included 3200 US adults (47.4% men; 0.8% American Indian or Alaska Native, 5.9% Asian, 12.0% Black or African American, 16.0% Hispanic or Latino, 63.6% White, and 1.9% other race; 34.8% with a college degree; and 36.7% with annual household income of ≥$75 000). Overall, 29.1% (95% CI, 27.0%-31.1%) of US adults were aware of synthetic cooling agent menthol-mimicking cigarettes, 36.9% (95% CI, 33.1%-40.8%) of those aware ever used these cigarettes, 24.7% (95% CI, 20.1%-29.3%) of those who ever used reported currently using these cigarettes, and 37.5% (95% CI, 33.4%-41.6%) of those not currently using were susceptible to using them. Black or African American (vs White), men (vs women), adults aged 60 years or younger (vs adults aged ≥61 years), adults who used menthol and nonmenthol cigarettes (vs adults who did not smoke cigarettes), and those who lived in states with a menthol cigarette ban (vs those who lived in states with no ban) had higher odds of awareness, ever use, current use, and/or susceptibility to these cigarettes. Considering a national menthol cigarette ban, 50.8% (95% CI, 42.8%-58.7%) of adults who smoked menthol cigarettes and were aware of menthol-mimicking cigarettes with a synthetic cooling agent reported likely to switch to these cigarettes. Ever using these cigarettes was associated with the likelihood of switching (adjusted odds ratio, 2.61 [95% CI, 1.20-5.68]).

**Conclusions and Relevance:**

In this survey study of US adults, a substantial proportion were aware of and had already experimented with synthetic cooling agent menthol-mimicking cigarettes. These products may serve as a substitute for menthol cigarettes and reduce the public health benefits of a menthol cigarette ban in promoting smoking cessation.

## Key Points

**Question:**

What geographic and demographic factors are related to the awareness and use of synthetic cooling agent menthol-mimicking cigarettes in the US?

**Findings:**

This cross-sectional survey study of 3200 US adults (≥21 years) found that a considerable proportion of adults were aware of and susceptible to and had experimented with or were currently using synthetic cooling agent menthol-mimicking cigarettes. Being Black or African American, being a man, being a younger adult, using menthol and nonmenthol cigarettes, and living in states with a menthol cigarette ban were associated with these outcomes.

**Meaning:**

Synthetic cooling agent menthol-mimicking cigarettes may serve as a substitute product in the presence of a ban on menthol cigarettes.

## Teaser

This survey study examines US adult awareness of, susceptibility to, use of, and intention to use synthetic cooling agent menthol-mimicking cigarettes.

## Introduction

Black or African American communities in the US are disproportionately bearing the negative health burden associated with commercial tobacco use.^1^ This is, in part, because of the targeted marketing of menthol cigarettes by the tobacco industry toward these communities.^1^ Consequently, whereas an estimated 33.8% of US adults who smoke cigarettes smoked menthol cigarettes in 2018 and 2019, 76.8% of Black adults who smoke cigarettes smoked menthol cigarettes in the same observation period.^2^ Menthol provides a cooling analgesic effect, which masks the irritation and harshness of cigarette smoke.^3^ This reduced irritation also allows for deeper inhalation, resulting in higher nicotine exposure.^3^ As expected, previous research found that menthol cigarettes promote smoking initiation and hinder smoking cessation.^4^ As a result, the addition of menthol to cigarettes was attributed to 10.1 million additional smokers, 378 000 premature deaths, and 3 million life-years lost from 1980 to 2018.^5^

To combat the detrimental health impact of menthol cigarettes, many countries, as well as states and localities in the US, have banned them from the marketplace. For example, menthol cigarettes were banned by the European Union in 2014, by Turkey in 2015, and by Canada in 2017.^6^ In the US, menthol cigarettes were banned in Massachusetts in 2020 and in California in 2022, as well as by cities in Colorado, Illinois, Maine, Minnesota, Ohio, Oregon, and Washington, DC.^7^ A recent systematic review and meta-analysis found that 24% of people who smoked menthol cigarettes quit smoking after a menthol cigarette ban.^8^ To circumvent such bans, cigarette companies have introduced synthetic cooling agent menthol-mimicking cigarettes into the marketplace.^9^ These cigarettes were manufactured by replacing menthol with the synthetic cooling agent WS-3,^10^ which is odorless and flavorless, and were marketed as “nonmenthol for menthol smokers.”^9^ This synthetic cooling agent can elicit a cooling perception in humans that is similar to that of menthol,^11^ which may prompt individuals who smoke menthol cigarettes to switch to these menthol-mimicking cigarettes instead of quitting smoking if a menthol cigarette ban is enacted nationwide.

Given the novelty of these products, the epidemiology associated with its awareness, experimentation, susceptibility, and current use is largely unknown. Additionally, if a nationwide menthol cigarette ban is enacted in the US, the likelihood that individuals who smoke menthol cigarettes switching to synthetic cooling agent menthol-mimicking cigarettes is unclear. Furthermore, it is unknown whether disclosing the cooling agent in these cigarettes will discourage switching from menthol cigarettes to menthol-mimicking cigarettes. Therefore, we conducted the first, to our knowledge, US national study to fill these knowledge gaps.

## Methods

### Study Population

This survey study (ie, the Cannabis, Alcohol, Nicotine, Discrimination and Lived Experience study) used many of the American Association for Public Opinion Research (AAPOR) Best Practices for Survey Research to design the survey and analyze the data. The study surveyed a national sample of adults in the US (≥21 years) who were part of the YouGov online panel^12^ between March and May 2024, with oversampling of individuals who use commercial tobacco, as well as racial and ethnic minority groups (American Indian or Alaska Native, Asian, Black or African American, and Hispanic or Latino individuals). YouGov uses a sample matching approach to provide nationally representative estimates. Briefly, YouGov used data from nationally representative surveys to draw a sample that met the sampling criteria of this study. A matching sample from the YouGov panelists, based on demographics and commercial tobacco use experiences, were recruited through email invitations. Sample matching has been previously demonstrated to achieve the sample level of representation as random-digit-dialing sampling after statistical weighting.^13^ Eligible panelists were invited to complete an online survey after providing informed consent and were compensated in accordance with YouGov policy. Individuals who provided inconsistent responses were excluded from the study, resulting in a predetermined sample size of 3200 (response rate, 73.5%). The National Institutes of Health institutional review board reviewed this study and determined it to be exempt from institutional review board review per 45 CFR §46.

### Measures

#### Awareness of and Experience With Synthetic Cooling Agent Menthol-Mimicking Cigarettes

Participants were shown pictures of Newport and Kool brand menthol- and nonmenthol-labeled cigarettes,^14^ with an explanation stating that these nonmenthol-labeled cigarettes had alternative cooling flavorings. They were then asked, “Before taking part in this study, have you heard of these ‘nonmenthol’ labelled cigarettes?” (with the response options: yes, no/don’t know/unsure). Those who answered “yes” were then asked, “What best describes your experience with ‘nonmenthol’ labelled cigarettes?” (with the response options: never used them, used before but not now, currently using some days, and currently using every day). Participants who answered “never used them” were considered as never using these cigarettes, whereas all other responses were considered as ever using these cigarettes. Additionally, participants who reported currently using synthetic cooling agent menthol-mimicking cigarettes daily and nondaily were considered as currently using these cigarettes (vs noncurrent using).

#### Susceptibility to and Likelihood of Switching to Synthetic Cooling Agent Menthol-Mimicking Cigarettes

Those who did not currently use synthetic cooling agent menthol-mimicking cigarettes at the time of survey were asked, “Are you curious about these ‘non-menthol’ labelled cigarettes?” “Do you think you will try these ‘non-menthol’ labelled cigarettes soon?” “If one of your best friends were to offer you these ‘non-menthol’ labelled cigarettes, would you use it?” (with the response options: definitely not, probably not, probably yes, and definitely yes). These measures were adapted from measures of susceptibility to cigarette smoking.^15^ Participants who answered “definitely not” to all 3 questions were categorized as nonsusceptible; otherwise, participants were categorized as susceptible. To test how disclosure of synthetic cooling agents may influence use intention if menthol cigarettes are banned in the US, participants were randomized into 2 conditions. In the first condition, participants were simply asked, “If menthol cigarettes are banned from the US, how likely are you to switch to these ‘non-menthol’ labelled cigarettes?” (with the response options: very unlikely, somewhat unlikely, unsure, somewhat likely, and very likely). In the second condition, participants were told, “In order to create the cooling sensation produced by menthol, cigarette companies added synthetic cooling chemicals, e.g., WS-3 and WS-23, to these ‘non-menthol’ labelled cigarettes.” They were then asked about their likelihood to switch to synthetic cooling agent menthol-mimicking cigarettes. Participants who answered “somewhat likely” and “very likely” were categorized as “Will switch if a menthol ban,” whereas others were categorized as “Will not switch.”

### Covariates

Participants self-reported their race and ethnicity (American Indian or Alaska Native, Asian, Black or African American, Hispanic or Latino, Middle Eastern or North African, Pacific Islander, multiracial or multiethnic, White, or other), age, gender (man, woman, or nonbinary), education, and annual household income. Racial and ethnic categories, including Middle Eastern or North African, Pacific Islander, multiracial or multiethnic, and other, were combined as “other” because of small sample sizes per these racial and ethnic groups. Education was categorized into “Up to high school/GED [General Education Development],” “some college,” and “college and above.” Annual household income was categorized as less than $25 000, $25 000 to $49 999, $50 000 to $74 999, and $75 000 or above. Participants also provided the states in which they resided at the time of survey. We categorized states into either with a menthol cigarette ban (California and Massachusetts) or without a menthol cigarette ban (all other states). Finally, participants reported their experience with cigarettes (with the response options: never used them, used before but not now, currently using some days, and currently using every day). Those who reported currently using cigarettes were asked whether they usually smoked menthol or nonmenthol cigarettes (with the response options: menthol, nonmenthol, and no usual type). Participants were categorized as “nonsmoking” if they reported not currently smoking cigarettes, as “current nonmenthol cigarette smoking” if they reported currently regularly smoking nonmenthol cigarettes, and as “current menthol cigarette smoking” if they reported currently regularly smoking menthol cigarettes or no usual type (ie, smoking menthol and nonmenthol cigarettes).

### Statistical Analysis

Data were weighted to be nationally representative. Descriptive statistics were used to summarize the population characteristics. Weighted prevalence of awareness of, ever use (among those aware), current use (among ever used), susceptibility (among noncurrent use), and likely to switch to synthetic cooling agent menthol-mimicking cigarettes were estimated overall and by covariate. Sequential weighted logistic regression models were used to estimate the association between covariates and synthetic cooling agent menthol-mimicking cigarettes (awareness, ever use, current use, or susceptibility). First, race and ethnicity, gender, age, and state menthol ban were included in the model as independent variables. Second, education and annual household income were added to the model. Third, current smoking behavior (ie, nonsmoking, nonmenthol cigarette smoking, or menthol cigarette smoking) was added to the model. To examine the associations with likelihood to switch to synthetic cooling agent menthol-mimicking cigarettes given a national menthol ban, a set of 3 weighted logistic regression models were conducted. First, race and ethnicity, gender, age, and state menthol ban were independent variables included in the model. Second, education and annual household income were added to the model. Third, ever used synthetic cooling agent menthol-mimicking cigarettes and randomized condition were added to the model. This approach was used to avoid adjusting for potential mediators when examining the association between an independent variable and an outcome. The statistical analysis was performed from July to September 2024, using SAS Enterprise, version 9.4 (SAS Institute). The threshold for significance was a 2-tailed *P* = .05.

## Results

The study sample included 3200 US adults (47.4% men; 0.8% American Indian or Alaska Native, 5.9% Asian, 12.0% Black or African American, 16.0% Hispanic or Latino, 63.6% White, and 1.9% other race; 34.8% with a college degree; and 36.7% with annual household income ≥$75 000, with fair equal distributions across the 4 age categories) (Table 1). Overall, 29.1% (95% CI, 27.0%-31.1%) of adults (≥21 years) were aware of synthetic cooling agent menthol-mimicking cigarettes. Results from multivariable logistic regression models showed that American Indian or Alaska Native and Black or African American adults (vs White adults; adjusted odds ratio [AOR] of 1.86 [95% CI, 1.35-2.58] and 1.94 [95% CI, 1.50-2.51], respectively), men (vs women; AOR, 1.40 [95% CI, 1.14-1.72]), adults 60 years or younger (vs adults aged ≥61 years ; AOR, 1.40 [95% CI, 1.03-1.90] to 2.72 [95% CI, 1.96-3.78]), and those residing in states with a menthol cigarette ban (vs those residing in states without a ban; AOR, 1.51 [95% CI, 1.11-2.04]) were more likely to be aware of synthetic cooling agent menthol-mimicking cigarettes, whereas nonbinary gender adults (vs women; AOR, 0.29 [95% CI, 0.10-0.83]) were less likely to be aware of these cigarettes (Table 1). Furthermore, adults who were currently smoking menthol or nonmenthol cigarettes were more likely than nonsmoking adults to be aware of these cigarettes (AOR of 1.69 [95% CI, 1.27-2.24] and 1.84 [95% CI, 1.42-2.39], respectively).

**Table 1.** Population Characteristics, Awareness of, Ever Use, Current Use, and Susceptible to Synthetic Cooling Agent Menthol-Mimicking Cigarettes and Their Associations (N = 3200)

Characteristic	Weighted % (No.)	Aware (n = 3200)		Ever (among aware [n = 1078])		Current (among ever [n = 493])		Susceptible (among noncurrent [n = 909])	
		Weighted % (No.)	AOR (95% CI)	Weighted % (No.)	AOR (95% CI)	Weighted % (No.)	AOR (95% CI)	Weighted % (No.)	AOR (95% CI)
Race and ethnicity									
American Indian or Alaska Native	0.8 (412)	40.5 (161)	1.86 (1.35-2.58)^a^	42.5 (81)	1.10 (0.66-1.82)	34.3 (30)	1.82 (0.86-3.87)	38.4 (58)	1.33 (0.70-2.53)
Asian	5.9 (431)	24.5 (107)	0.75 (0.55-1.03)	29.2 (41)	0.52 (0.29-0.94)^a^	24.0 (17)	0.96 (0.43-2.16)	35.7 (37)	1.03 (0.53-1.98)
Black or African American	12.0 (540)	42.1 (248)	1.94 (1.50-2.51)^a^	34.9 (116)	0.79 (0.51-1.21)	18.5 (32)	0.77 (0.41-1.44)	43.0 (115)	1.59 (1.01-2.52)^a^
Hispanic or Latino	16.0 (471)	33.9 (168)	1.17 (0.88-1.55)	33.4 (63)	0.63 (0.39-1.02)	31.1 (25)	1.25 (0.62-2.54)	44.5 (68)	2.78 (0.81-2.23)
White	63.6 (1255)	25.9 (374)	1 [Reference]	39.8 (186)	1 [Reference]	24.4 (63)	1 [Reference]	33.6 (140)	1 [Reference]
Other race^b^	1.9 (91)	21.7 (20)	0.62 (0.30-1.31)	17.7 (6)	0.27 (0.08-0.86)^a^	NA	NA	38.1 (9)	1.05 (0.54-4.50)
Gender									
Nonbinary	1.2 (44)	12.4 (9)	0.29 (0.10-0.83)^a^	NA	NA	NA	NA	NA	NA
Woman	51.4 (1615)	26.0 (497)	1 [Reference]	33.5 (206)	1 [Reference]	24.7 (74)	1 [Reference]	26.5 (153)	1 [Reference]
Man	47.4 (1541)	32.9 (572)	1.40 (1.14-1.72)^a^	40.0 (285)	1.27 (0.90-1.80)	24.4 (93)	0.84 (0.50-1.41)	47.3 (270)	2.76 (1.88-4.05)^a^
Age, y									
21-30	18.3 (575)	39.4 (240)	2.72 (1.96-3.78)^a^	45.1 (120)	4.14 (2.22-7.71)^a^	22.9 (42)	4.49 (1.23-16.35)^a^	59.9 (128)	13.60 (6.73-27.47)^a^
31-45	26.5 (1001)	36.9 (434)	2.44 (1.83-3.26)^a^	49.1 (250)	5.02 (2.80-9.00)^a^	32.9 (98)	7.10 (2.06-24.46)^a^	48.4 (196)	8.79 (4.56-16.95)^a^
46-60	24.2 (878)	25.5 (252)	1.40 (1.03-1.90)^a^	27.0 (90)	1.94 (1.02-3.67)^a^	16.1 (23)	2.69 (0.69-10.42)	24.9 (79)	3.07 (1.55-6.07)^a^
≥60	31.0 (746)	19.0 (152)	1 [Reference]	17.3 (33)	1 [Reference]	5.8 (5)	1 [Reference]	10.3 (24)	1 [Reference]
State menthol ban									
Yes	11.6 (459)	37.6 (177)	1.51 (1.11-2.04)^a^	41.0 (88)	1.27 (0.81-1.99)	33.2 (33)	1.52 (0.81-2.84)	41.5 (77)	1.13 (0.69-1.85)
No	88.4 (2741)	28.0 (901)	1 [Reference]	36.2 (405)	1 [Reference]	23.0 (135)	1 [Reference]	36.9 (350)	1 [Reference]
Education									
Up to high school or GED	35.7 (1219)	30.0 (434)	1.02 (0.78-1.33)	40.0 (215)	1.69 (1.08-2.64)^a^	22.5 (64)	0.94 (0.48-1.83)	35.8 (173)	0.91 (0.56-1.49)
Some college	29.5 (934)	27.7 (293)	0.95 (0.72-1.26)	37.2 (125)	1.47 (0.92-2.35)	17.6 (41)	0.67 (0.34-1.33)	37.1 (109)	1.06 (0.64-1.74)
College or above	34.8 (1047)	29.3 (351)	1 [Reference]	33.5 (153)	1 [Reference]	33.7 (63)	1 [Reference]	39.7 (145)	1 [Reference]
Annual household income, $									
<25 000	23.7 (861)	31.3 (303)	0.97 (0.73-1.30)	39.3 (146)	1.12 (0.67-1.88)	20.2 (46)	0.46 (0.22-0.96)^a^	45.1 (131)	1.75 (1.00-3.04)^a^
25 000-49 999	20.1 (675)	29.5 (235)	1.09 (0.81-1.47)	37.4 (101)	1.21 (0.74-1.97)	15.5 (28)	0.34 (0.16-0.73)^a^	37.1 (96)	1.67 (1.01-2.78)^a^
50 000-74 999	19.5 (590)	25.2 (175)	0.82 (0.61-1.10)	38.4 (84)	1.20 (0.74-1.95)	18.6 (22)	0.44 (0.21-0.94)^a^	40.7 (73)	1.54 (0.86-2.74)
≥75 000	36.7 (1065)	29.4 (362)	1 [Reference]	34.6 (161)	1 [Reference]	36.8 (72)	1 [Reference]	31.0 (126)	1 [Reference]
Current smoking									
Nonmenthol	8.7 (502)	45.2 (245)	1.84 (1.42-2.39)^a^	63.4 (173)	3.41 (2.22-5.23)^a^	41.5 (73)	12.49 (6.19-25.21)^a^	83.5 (141)	13.24 (6.56-26.71)^a^
Menthol	8.8 (459)	36.7 (180)	1.69 (1.27-2.24)^a^	64.0 (119)	4.19 (2.61-6.71)^a^	58.8 (72)	25.59 (11.37-57.62)^a^	61.2 (67)	6.18 (3.48-10.98)^a^
Nonsmoking	82.5 (2239)	26.6 (653)	1 [Reference]	28.2 (201)	1 [Reference]	6.4 (23)	1 [Reference]	29.1 (219)	1 [Reference]

Abbreviations: AOR, adjusted odds ratio; GED, General Education Development; NA, not available (ie, suppressed).

^a^ Estimates are statistically significant (*P* < .05).

^b^ Includes those who self-identified as Middle Eastern or North African, Pacific Islander, multiracial or multiethnic, or other.

Among adults who were aware of synthetic cooling agent menthol-mimicking cigarettes (n = 1078), 36.9% (95% CI, 33.1%-40.8%) had ever used them. Findings from multivariable logistic regression models showed that adults 60 years or younger (vs those aged ≤61 years; AOR, 1.94 [95% CI, 1.02-3.67] to 4.14 [95% CI, 2.22-7.71]), those with up to a high school education or GED (vs those with a college degree or above; AOR, 1.69 [95% CI, 1.08-2.64]), and those who were currently smoking menthol or nonmenthol cigarettes (vs those who were nonsmoking; AOR of 4.19 [95% CI, 2.61-6.71] and 3.41 [95% CI, 2.22-5.23], respectively) were more likely to have ever used these cigarettes (Table 1). In contrast, Asian adults (AOR, 0.52 [95% CI, 0.29-0.94]) and adults from other race and ethnicity groups (AOR, 0.27 [95% CI, 0.08-0.86]) were less likely than White adults to have ever used these cigarettes.

Among adults who ever used synthetic cooling agent menthol-mimicking cigarettes (n = 493), 24.7% (95% CI, 20.1%-29.3%) were currently using these cigarettes at least some days. Results from multivariable logistic regression models revealed that adults 60 years or younger (vs those aged ≥61 years; AOR, 4.49 [95% CI, 1.23-16.35] to 7.10 [95% CI, 2.06-24.46]) and those currently smoking menthol or nonmenthol cigarettes (vs those who ere nonsmoking; AOR of 25.59 [95% CI, 11.37-57.62] and 12.49 [95% CI, 6.19-25.21], respectively) were more likely to have ever used synthetic cooling agent menthol-mimicking cigarettes (Table 1). It is worth noting that adults who currently smoked menthol cigarettes were much more likely than those who smoked nonmenthol cigarettes to be currently using synthetic cooling agent menthol-mimicking cigarettes. In contrast, adults with a lower annual household income (vs those with an annual household income ≥$75 000; AOR, 0.34 [95% CI, 0.16-0.73] to 0.46 [95% CI, 0.22-0.96]) were less likely to be currently using these cigarettes.

Among adults who previously used synthetic cooling agent menthol-mimicking cigarettes (n = 909), 37.5% (95% CI, 33.4%-41.6%) were susceptible to using them. Black or African American adults (vs White adults; AOR, 1.59 [95% CI, 1.01-2.52]), men (vs women; AOR, 2.76 [95% CI, 1.88-4.05]), young adults (vs those aged ≥61 years; AOR, 3.07 [95% CI, 1.55-6.07] to 13.60 [95% CI, 6.73-27.47]), those with a less than $50 000 annual household income (vs those with ≥$75 000; AOR, 1.67 [95% CI, 1.01-2.78] to 1.75 [95% CI, 1.00-3.04]), and those currently smoking menthol or nonmenthol cigarettes (vs those who were nonsmoking; AOR of 6.18 [95% CI, 3.48-10.98] and 13.24 [95% CI, 6.56-26.71], respectively) were more likely to be susceptible to synthetic cooling agent menthol-mimicking cigarette use, as shown in multivariable logistic regression models (Table 1).

Table 2 shows the results from multivariable logistic regression models for switching to synthetic cooling agent menthol-mimicking cigarettes if there is a menthol cigarette ban in the US among adults who currently smoked menthol cigarettes and were aware of these cigarettes (n = 245). Overall, 50.8% (95% CI, 42.8%-58.7%) of these individuals stated that they would switch to synthetic cooling agent menthol-mimicking cigarettes. Black or African American and Hispanic or Latino adults (vs White adults; AOR of 0.46 [95% CI, 0.22-0.96] and 0.31 [95% CI, 0.11-0.84], respectively) and those with annual household income of $50 000 to $74 999 (vs ≥$75 000; AOR, 0.29 [95% CI, 0.10-0.81]) were less likely to switch to these cigarettes. In contrast, those who had ever used synthetic cooling agent menthol-mimicking cigarettes had more than twice the odds than those who never used these cigarettes to switch to these cigarettes if a menthol cigarette ban were enacted in the US (AOR, 2.61 [95% CI, 1.20-5.68]). Disclosing the chemical cooling agent was not associated with intention to switch to synthetic cooling agent menthol-mimicking cigarettes (AOR, 1.15 [95% CI, 0.57-2.33]).

**Table 2.** Characteristics of US Adults Who Smoke Menthol Cigarettes and Were Aware of Synthetic Cooling Agent Menthol-Mimicking Cigarettes and Their Associations With Switching to Menthol-Mimicking Cigarettes if Menthol Cigarettes Are Banned in the US (N = 245)

Characteristic	Weighted % (No.)	Switching to menthol-mimicking cigarettes if menthol cigarettes are banned	
		Weighted % (No.)	AOR (95% CI)
Race and ethnicity			
American Indian or Alaska Native	1.2 (37)	36.6 (16)	0.40 (0.16-1.03)
Asian	1.5 (11)	NA	NA
Black	22.2 (77)	38.9 (34)	0.46 (0.22-0.96)^a^
Hispanic or Latino	14.1 (28)	31.4 (9)	0.31 (0.11-0.84)^a^
White	58.0 (87)	59.5 (52)	1 [Reference]
Other^b^	2.9 (5)	NA	NA
Gender			
Man	63.8 (103)	50.3 (72)	0.94 (0.47-1.86)
Nonbinary	0 (1)	NA	NA
Woman	36.2 (69)	51.7 (48)	1 [Reference]
Age, y			
21-30	36.1 (65)	51.1 (33)	0.88 (0.19-4.07)
31-45	42.7 (123)	53.3 (63)	1.02 (0.24-4.32)
46-60	15.0 (44)	42.4 (19)	0.71 (0.14-3.52)
≥61	6.2 (13)	51.5 (5)	1 [Reference]
State menthol ban			
Yes	16.9 (38)	57.3 (23)	1.33 (0.50-3.55)
No	83.1 (207)	49.5 (97)	1 [Reference]
Education			
Up to high school or GED	42.2 (118)	51.8 (57)	1.56 (0.59-4.12)
Some college	24.2 (58)	45.0 (26)	0.36 (0.51-3.61)
College or above	33.6 (69)	53.7 (37)	1 [Reference]
Annual household income, $			
<25 000	23.2 (78)	47.6 (31)	0.47 (0.16-1.22)
<50 000	21.7 (53)	48.8 (25)	0.48 (0.17-1.36)
<75 000	23.1 (49)	36.1 (20)	0.29 (0.10-0.81)^a^
≥75 000	32.0 (64)	65.1 (43)	1 [Reference]
Ever used menthol-mimicking cigarettes			
Yes	63.4 (173)	58.3 (95)	2.61 (1.20-5.68)^a^
No	36.6 (72)	37.8 (25)	1 [Reference]
Experimental condition: named cooling agent			
Yes	53.1 (127)	52.5 (63)	1.15 (0.57-2.33)
No	46.9 (118)	48.8 (57)	1 [Reference]

Abbreviations: AOR, adjusted odds ratio; GED, General Education Development; NA, not available (ie, same size too small to disclose or estimate an association).

^a^ Estimates are statistically significant (*P* < .05).

^b^ Other race includes those who self-identified as Middle Eastern or North African, Pacific Islander, multiracial or multiethnic, or other.

## Discussion

The tobacco industry introduced synthetic cooling agent menthol-mimicking cigarettes into the US marketplace as menthol cigarette bans were gaining momentum in localities and states. As expected, respondents living in states with a menthol cigarette ban were more likely to be aware of these cigarettes than those living in states without such a ban, suggesting that the ban may have increased their receptivity to synthetic cooling agent menthol-mimicking cigarette advertising. However, even in states without a menthol cigarette ban, close to 3 in 10 adults were aware of synthetic cooling agent menthol-mimicking cigarettes. Furthermore, over one-third of those who were aware of these cigarettes had experimented with them, almost one-fourth of those who experimented with them were currently using them, and over one-third of those who were not currently using them were susceptible to using them. These findings further suggest that synthetic cooling agent menthol-mimicking cigarettes are available and promoted nationwide. Because synthetic cooling agent menthol-mimicking cigarettes contain a cooling agent (WS-3) that is known to activate the same sensory receptors as menthol,^11^ which reduces the harshness of cigarette smoke, these cigarettes could promote cigarette smoking initiation just like menthol cigarettes. Additionally, given that US adults who were Black or African American, men, younger individuals, and smoked cigarettes (especially menthol cigarettes) were more likely to be aware, susceptible, experimenting, and/or currently using synthetic cooling agent menthol-mimicking cigarettes, these products could undermine the public health and health equity benefits of a menthol cigarette ban given that some of these groups also have the highest prevalence of current menthol cigarette use.^16^ Moreover, although it is expected that, among US adults who previously used synthetic cooling agent menthol-mimicking cigarettes, those who smoked menthol cigarettes currently would be more susceptible to future synthetic cooling agent menthol-mimicking cigarette use than those who did not smoke, given the familiarity of the cooling sensation provided by the synthetic cooling agents. It is noteworthy that US adults who smoked nonmenthol cigarettes were also more susceptible to future synthetic cooling agent menthol-mimicking cigarette use than those who did not smoke cigarettes. Perhaps, adults who do not smoke menthol cigarettes are interested in the reduced harshness without the menthol flavor. Future research needs to understand how synthetic cooling agent menthol-mimicking cigarettes may affect the smoking behaviors of adults who smoke nonmenthol cigarettes.

We found that, among US adults who currently smoked menthol cigarettes and were aware of synthetic cooling agent menthol-mimicking cigarettes, half indicated that they were likely to switch to these cigarettes if menthol cigarettes were banned in the US. Moreover, experimentation with synthetic cooling agent menthol-mimicking cigarettes also increases the odds of switching from menthol cigarettes to these cigarettes, with Black or African American and Hispanic adults showing lower odds of switching. However, among those who previously tried synthetic cooling agent menthol-mimicking cigarettes, Black or African American (vs White) men (vs women) were more likely to be susceptible to future use. Because previous studies showed that about one-fourth of individuals who smoke menthol cigarettes would quit smoking altogether if menthol cigarettes were banned, the availability of synthetic cooling agent menthol-mimicking cigarettes may reduce the proportion of adults who smoke menthol cigarettes quitting smoking because of menthol cigarette bans. However, it is less clear how synthetic cooling agent menthol-mimicking cigarettes would affect disparities in harm associated with menthol cigarettes.

Interestingly, disclosing the synthetic cooling agent used in synthetic cooling agent menthol-mimicking cigarettes did not discourage switching to these cigarettes. Perhaps it was because all respondents were told that these cigarettes contain cooling agents during the introduction of this section of the survey. Therefore, further disclosing the name of the cooling agent did not change switching intention. It is also possible that respondents did not associate the cooling agent with any health risks. Explaining to the public that WS-3 is used in shaving cream may yield a different response. Future research needs to investigate how to communicate the potential harm of synthetic cooling agent menthol-mimicking cigarettes to individuals and to the public.

It is worth noting that synthetic cooling agents are also present in other tobacco products that are subjected to flavor bans. For example, Leventhal and colleagues reported that e-cigarettes with cooling flavors contain synthetic cooling agents such as WS-3 and WS-23.^17^ Tackett and colleagues conducted an experiment with adults who use cigarettes and/or e-cigarettes and found that e-cigarettes with these cooling agents, compared with those without, were rated higher in liking, willingness to use, and smoothness.^18^ Synthetic cooling agents are also present in oral nicotine pouches, and manufacturers have been marketing these products as “flavor-ban approved.”^19^ Therefore, future menthol- and flavor-ban policies need to discern a legal standard that would prohibit any flavorings (eg, based on user-reported sensation or neuroreceptor activation).

### Limitations

Despite this being the first US national study, to our knowledge, to examine the epidemiology of synthetic cooling agent menthol-mimicking cigarette use, this study has some limitations. Because participants were recruited from an online panel, individuals who do not have access to the internet are not eligible to participate in the study. However, sample matching minimizes the bias resulting from this exclusion. We also only included adults at least 21 years of age. Therefore, our findings may not be generalizable to those younger than 21 years. Furthermore, we only considered state-level menthol cigarette bans in this analysis. There are localities that passed menthol cigarette bans located in states without such a ban. Participants in these localities were classified as living in a state without a menthol ban. Last, because many people may not be familiar with synthetic cooling agent menthol-mimicking cigarettes, there is a possibility of recall error. However, we provided pictures and descriptions of these cigarettes in the survey alongside menthol cigarettes to help participants distinguish between them.

## Conclusions

This survey study found that many adults in the US were aware of, are susceptible to, have experimented with, and were currently using synthetic cooling agent menthol-mimicking cigarettes, particularly in states with a menthol cigarette ban, as well as among younger individuals, men, and/or those who are Black or African American. Many adults who smoked menthol cigarettes indicated that they would switch to synthetic cooling agent menthol-mimicking cigarettes if a nationwide menthol cigarette ban were to be enacted. Further research is needed to investigate how to communicate the potential harm of synthetic cooling agent menthol-mimicking cigarettes to individuals and public health. Future flavor-ban policies should also explore a more comprehensive definition for flavors to prevent the tobacco industry from circumventing these policies.

## Notes

Supplement.
Data Sharing Statement
