# Supplementary material for: Knowledge and Use of Menthol-Mimicking Cigarettes Among Adults in the US
Source: JAMA Netw Open. 2025 Jan 14;8(1):e2454608. doi: 10.1001/jamanetworkopen.2024.54608 (PMC11733694; doi:10.1001/jamanetworkopen.2024.54608)
Supplement: Supplement. — Data Sharing Statement [file jamanetwopen-e2454608-s001.pdf]

Data Sharing Statement

Choi. Knowledge and Use of Menthol-Mimicking Cigarettes Among Adults in the US. *JAMA Netw Open*. Published January 14, 2025. doi:10.1001/jamanetworkopen.2024.54608

Data
Data available: Yes
Data types: Deidentified participant data, Data dictionary
How to access data: Data requests should be sent to kelvin.choi@nih.gov.
When available: With publication

Supporting Documents
Document types: None

Additional Information
Who can access the data: Researchers with an approved proposal.
Types of analyses: Academic publications.
Mechanisms of data availability: With a signed data use agreement.
